# ADP-ribose/TRPM2-mediated Ca^2+^ signaling is essential for cytolytic degranulation and antitumor activity of natural killer cells

**DOI:** 10.1038/srep09482

**Published:** 2015-03-25

**Authors:** So-Young Rah, Jae-Yong Kwak, Yun-Jo Chung, Uh-Hyun Kim

**Affiliations:** 1 Department of Biochemistry, Chonbuk National University Medical School, Jeonju, Republic of Korea; 2 National Creative Research Laboratory for Ca^2+^ signaling Network, Chonbuk National University Medical School, Jeonju, Republic of Korea; 3 Institute of Cardiovascular Research, Chonbuk National University Medical School, Jeonju, Republic of Korea; 4 Division of Hematology and Oncology, Department of Internal Medicine, Chonbuk National University Medical School, Jeonju, Republic of Korea; 5 Research Institute of Clinical Medicine, Chonbuk National University Medical School, Jeonju, Republic of Korea

## Abstract

Natural killer (NK) cells are essential for immunosurveillance against transformed cells. Transient receptor potential melastatin 2 (TRPM2) is a Ca^2+^-permeable cation channel gated by ADP-ribose (ADPR). However, the role of TRPM2-mediated Ca^2+^ signaling in the antitumor response of NK cells has not been explored. Here, we show that ADPR-mediated Ca^2+^ signaling is important for cytolytic granule polarization and degranulation but not involved in target cell recognition by NK cells. The key steps of this pathway are: 1) the activation of intracellular CD38 by protein kinase A following the interaction of the NK cell with a tumor cell results in the production of ADPR, 2) ADPR targets TRPM2 channels on cytolytic granules, and 3) TRPM2-mediated Ca^2+^ signaling induces cytolytic granule polarization and degranulation, resulting in antitumor activity. NK cells treated with 8-Br-ADPR, an ADPR antagonist, as well as NK cells from *Cd38^−/−^* mice showed reduced tumor-induced granule polarization, degranulation, granzyme B secretion, and cytotoxicity of NK cells. Furthermore, TRPM2-deficient NK cells showed an intrinsic defect in tumoricidal activity. These results highlight CD38, ADPR, and TRPM2 as key players in the specialized Ca^2+^ signaling system involved in the antitumor activity of NK cells.

Natural killer (NK) cells are large globular lymphocytes that represent our innate immune response against virally-infected or transformed cells^1^,^2^. After NK cells recognize tumor cells, NK cell receptors are activated, which likely aids the formation of an immunological synapse, towards which cytolytic granules containing perforin and granzymes, and the microtubule organizing center of NK cells are polarized^3^,^4^. After the cytolytic granules fuse with the plasma membrane through the degranulation process, the secreted perforin forms pores in the plasma membrane of the tumor cells. Serine protease granzyme B enters tumor cells through perforin and induces caspase-dependent and independent apoptotic cancer cell death^5^,^6^. Intracellular Ca^2+^ mobilization is required for target cell adhesion, granule polarization, and the degranulation process of NK cells, which are necessary in order to display their natural cytotoxicity^7^. Prior study suggests that cytotoxic lymphocyte degranulation and target cell lysis are Ca^2+^-dependent through STIM1/ORAI1-mediated calcium influx^8^. Recently, it has also been reported that exocytotic granules are themselves acidic Ca^2+^ stores, and a more target-specific Ca^2+^-mobilizing messenger, such as nicotinic acid adenine dinucleotide phosphate (NAADP), has been identified as being critical for the release of Ca^2+^ from exocytolytic granules via their cognate two-pore channels (TPCs), leading to cytolytic activity in cytotoxic T lymphocytes (CTLs)^9^. However, the precise mechanism by which Ca^2+^ signals interplay in cytolytic granule exocytosis and the killing of NK cells has remained unclear.

Transient receptor potential melastatin 2 (TRPM2) is a Ca^2+^-permeable nonselective cation channel localized at the lysosomal membrane as well as the plasma membrane^10^,^11^,^12^,^13^,^14^,^15^,^16^, and TRPM2-mediated Ca^2+^ signaling is involved in innate immunity^17^. TRPM2 channels are opened through the binding of intracellular ADP-ribose (ADPR) and can be synergistically activated by the presence of cyclic ADP-ribose (cADPR), NAADP, hydrogen peroxide (H_2_O_2_), and Ca^2+^ 18,19,20,21.

CD38 is a multifunctional enzyme that catalyzes the synthesis of Ca^2+^-mobilizing second messengers, cADPR and NAADP, from β-nicotinamide adenine dinucleotide (β-NAD^+^) and its phosphate form (β-NADP^+^), respectively^22^,^23^,^24^. NAADP and cADPR are further converted to ADP-ribose 2′-phosphate and ADPR, respectively^22^,^23^,^25^. CD38 has long been known to trigger cytotoxic responses and release granzymes in activated NK cells^26^, but the precise mechanisms by which CD38 mediates cytolytic activity have remained obscure. Interleukin 2 (IL-2)-activated NK cells are more lytic to target cells than resting NK cells, suggesting that IL-2 induces the *de novo* expression of proteins that act between CD38 and the lytic machinery in NK cells^27^.

In this study, we explored the possibility that ADPR may affect the antitumor effects of NK cells by modulating [Ca^2+^] via the TRPM2 channel. We have identified a novel mechanism for antitumor function of NK cells, in which ADPR produced by CD38 and TRPM2-dependent Ca^2+^ release from acidic Ca^2+^ stores result in cytolytic granule polarization and degranulation. These findings may help to better understand the regulation of NK cell cytotoxicity and offer a therapeutic strategy for enhancing the antitumor function of NK cells.

## Results

### NK cells from TRPM2-deficient mice have an intrinsic defect in antitumor activity

**Figure 1:**
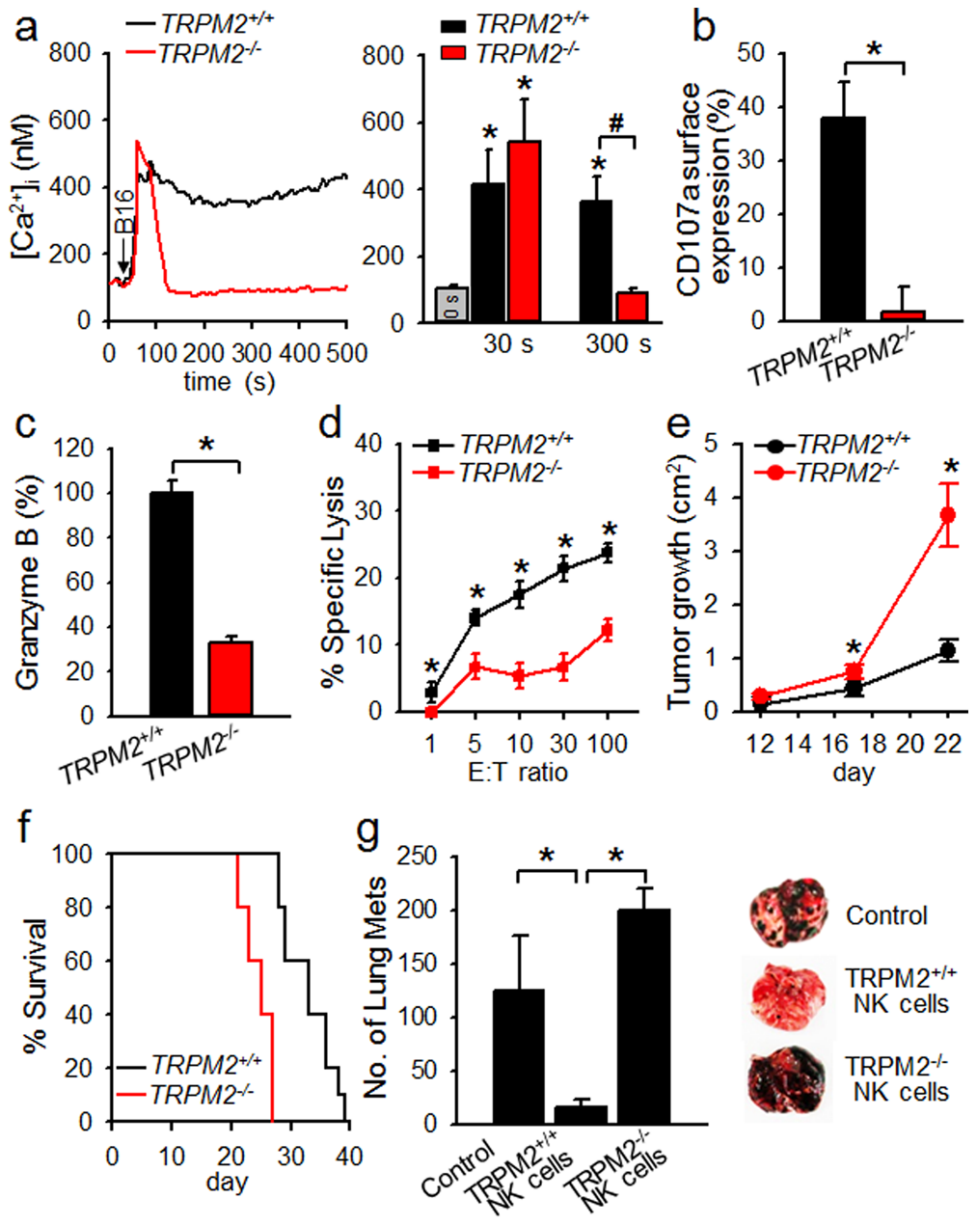
Critical role of TRPM2 for cytotoxicity of NK cells against tumor cells via Ca^2+^-dependent degranulation. (a) Tumor cell-induced Ca^2+^ signals in NK cells from *TRPM2^+/+^* or *TRPM2^−/−^* mice. Arrow indicates the time of addition of B16F10 tumor cells. Data are mean ± SEM of three independent experiments. *P < 0.001 vs basal; ^#^*P* < 0.05. (b) Impairment of degranulation in *TRPM2^−/−^* NK cells upon stimulation with B16F10 cells. *TRPM2^+/+^* or *TRPM2^−/−^* NK cells were stimulated with target cells for 2 h at 37°C and then stained with FITC-conjugated anti-CD107a mAb and PE-conjugated anti-NK1.1 mAb. NK cells were gated on forward scatter/side scatter characteristics. (c) Reduced granzyme B release in tumor cell-stimulated *TRPM2^−/−^* NK cells. The amount of granzyme B released into the media was measured by ELISA after incubation with NK cells and B16F10 cells for 30 min. (d) Decrease in cytolytic activity against B16F10 cells in *TRPM2^−/−^* NK cells. *TRPM2^+/+^* or *TRPM2^−/−^* NK cells were assessed for cytolytic activity against B16F10 target cells in a 4-h calcein-release assay. Data shown in *b*, *c*, and *d* are representative of three independent experiments. *P < 0.001. (e) B16F10 cells (1 × 10^5^) were injected into the flanks of *TRPM2^+/+^* and *TRPM2^−/−^* mice (n = 10 per cohort), and tumor growth was monitored. *P < 0.001. (f) Kaplan-Meier plot of *TRPM2^+/+^* and *TRPM2^−/−^* recipient mice after s.c. injection with B16F10 cells (1 × 10^5^) (n = 10 per cohort). (g) Antitumor effect of *TRPM2^+/+^* or *TRPM2^−/−^* NK cells in the lung metastasis model of B16F10 cells. *TRPM2^−/−^* mice (n = 4 per cohort) were injected i.v. with 1 × 10^5^ B16F10 melanoma cells. IL-2 activated NK cells (2 × 10^6^ cells) from *TRPM2^+/+^* or *TRPM2^−/−^* mice were injected i.v. into B16F10-bearing *TRPM2^−/−^* mice 2 d later. Lungs were harvested 14 d after and fixed with 4% paraformaldehyde, and metastatic nodules were counted. Representative images of lungs are shown. *P < 0.01.

To evaluate the possibility that TRPM2-mediated Ca^2+^ signaling is required for the antitumor effector function of NK cells, we first examined the tumor-induced Ca^2+^ change in NK cells from *TRPM2^+/+^* and *TRPM2^−/−^* mice. We noticed robust Ca^2+^ signals in both *TRPM2^+/+^* and *TRPM2^−/−^* NK cells upon contact with B16F10 cells, a melanoma tumor cell line. However, *TRPM2^+/+^* NK cells were distinct from *TRPM2^−/−^* NK cells in their ability to sustain the Ca^2+^ signals. *TRPM2^+/+^* NK cells exhibited a rapid initial increase, after which the elevated levels remained for the duration of our measurement (500 s). In contrast, *TRPM2^−/−^* NK cells were not able to sustain the initial intracellular [Ca^2+^] ([Ca^2+^]_i_) rise (Fig. 1a; 31.5% of area under curve (AUC) of Ca^2+^ trace in *TRPM2^+/+^* NK cells). As [Ca^2+^]_i_ increase is important to the degranulation of NK cells^7^, we investigated whether TRPM2 was involved in degranulation activity. We compared the surface expression of CD107a in *TRPM2^+/+^* and *TRPM2^−/−^* NK cells following stimulation with B16F10. B16F10-induced degranulation was absent in *TRPM2^−/−^* NK cells (Fig. 1b). Tumor cell-induced granzyme B secretion and cytotoxicity were also completely absent in *TRPM2^−/−^* NK cells (Fig. 1c and d). We further evaluated the ability of TRPM2 to protect against B16F10 tumor growth *in vivo*. Consistent with our finding that *TRPM2^−/−^* NK cells have decreased cytolytic activity (Fig. 1d), we observed a defect in the ability of TRPM2 deficient mice to control B16F10 tumor formation and their consequent survival rates (Fig. 1e and f). These results suggest that TRPM2 channel-mediated Ca^2+^ signals are critical for the antitumor effect of NK cells.

To assess whether the defect in B16F10 tumor control was due to NK cell dysfunction, we adoptively transferred IL-2 activated NK cells from *TRPM2^+/+^* and *TRPM2^−/−^* mice into B16F10 tumor-bearing *TRPM2^−/−^* mice. Following B16F10 tumor cell injection, a markedly higher number of metastatic cells were observed in *TRPM2^−/−^* NK cell-treated mice than in *TRPM2^+/+^* NK cell-treated mice (Fig. 1g). These findings suggest that TRPM2-deficient NK cells have an intrinsic defect in antitumor activity.

### CD38 is associated with the granule polarization and degranulation process of NK cells

**Figure 2:**
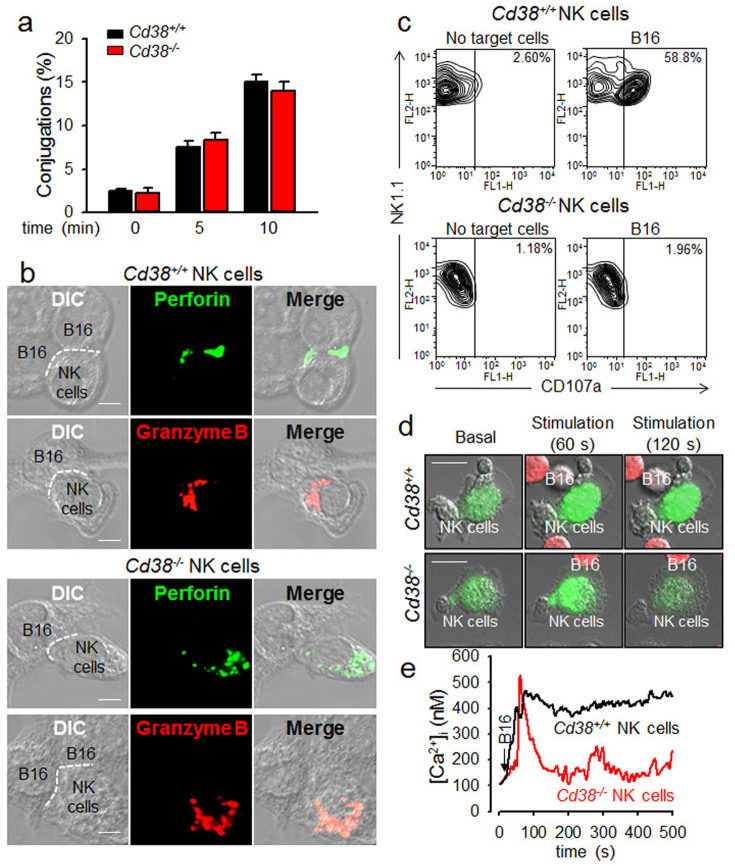
Tumor cell-induced NK cell granule polarization and degranulation requires CD38-mediated Ca^2+^ signals. (a) *Cd38^+/+^* or *Cd38^−/−^* NK cells stimulated with B16F10 tumor cells. The percentage of conjugation formation at indicated time was analyzed by flow cytometry. Data are mean ± SEM of three independent experiments. (b) *Cd38^−/−^* NK cells were inhibited tumor-triggered translocation of perforin and granzyme B towards immunological synapses. *Cd38^+/+^* or *Cd38^−/−^* NK cells were stimulated with B16F10 cells at 1:1 ratio at 37°C for 20 min, and then stained with perforin or granzyme B. The dashed line indicates the immunological synapse. (Scale bar, 5 μm). All images were representative of at least three independent experiments. (c) Degranulation of *Cd38^+/+^* or *Cd38^−/−^* NK cells upon stimulation with B16F10 cells for 2 h at 37°C. Shown is a representative of three independent experiments. (d and e) Ca^2+^ signals in *Cd38^+/+^* or *Cd38^−/−^* NK cells upon stimulation with B16F10 cells. NK cells were loaded with Fluo-4 AM for 40 min at 37°C and Ca^2+^ levels in NK cells were measured following treatment with B16F10 cells labeled with cell tracker orange CMRA (red). Arrow indicates the time of addition of B16F10 cells. (Scale bars, 10 μm). Shown is a representative of three independent experiments.

The TRPM2 channel can be synergistically opened by various Ca^2+^-mobilizing second messengers, such as ADPR, cADPR, or NAADP, all of which are produced by CD38^18^,^19^,^23^,^24^. NK cell-mediated tumor clearance is composed of target cell adhesion, granule polarization, and degranulation, and CD38 is known to trigger cytotoxic responses as a receptor in NK cells^3^,^26^. Therefore, we further examined whether mouse *Cd38^+/+^* NK cells and *Cd38^−/−^* NK cells differed in their ability to induce tumor cell adhesion when co-cultured with B16F10 cells. Time-dependent increases in the number of conjugates between NK and tumor cells were observed in both *Cd38^+/+^* and *Cd38^−/−^* NK cells with no significant differences (Fig. 2a).

We next investigated whether tumor cell-induced NK cell granule polarization was affected by CD38 by analyzing the translocation of cytolytic granules containing perforin and granzyme B. In the absence of target cells, perforin and granzyme B displayed dispersed granules in NK cells. Upon conjugation with B16F10, perforin and granzyme B were translocated towards the immunological synapse area between *Cd38^+/+^* NK cells and B16F10 cells. However, such tumor cell-induced translocation of perforin and granzyme B was not observed in *Cd38^−/−^* NK cells (Fig. 2b). To address the question of whether the degranulation activity of NK cells was affected by CD38, we compared the surface expression of CD107a in *Cd38^+/+^* and *Cd38^−/−^* NK cells following stimulation with B16F10. B16F10 induced strong degranulation in *Cd38^+/+^* NK cells but not in *Cd38^−/−^* NK cells (Fig. 2c). These results demonstrate that CD38 is involved in the granule polarization and cytolytic degranulation process of NK cell during contact with tumor cells, but not in tumor cell adhesion.

We next investigated whether the tumor-induced [Ca^2+^]_i_ increase was dependent on CD38. Similar to *TRPM2^−/−^* NK cells, *Cd38^−/−^* NK cells displayed the tumor-induced initial rise in [Ca^2+^]_i_, but failed to maintain the elevated levels (Fig. 2d and e; 36.1% of AUC of Ca^2+^ trace in *Cd38^+/+^* NK cells). These results demonstrate that CD38-mediated Ca^2+^ signaling may be involved in the granule polarization and cytolytic degranulation process of NK cell during contact with tumor cells.

### Cytolytic granule polarization of NK cells requires ADPR-mediated Ca^2+^ signaling

**Figure 3:**
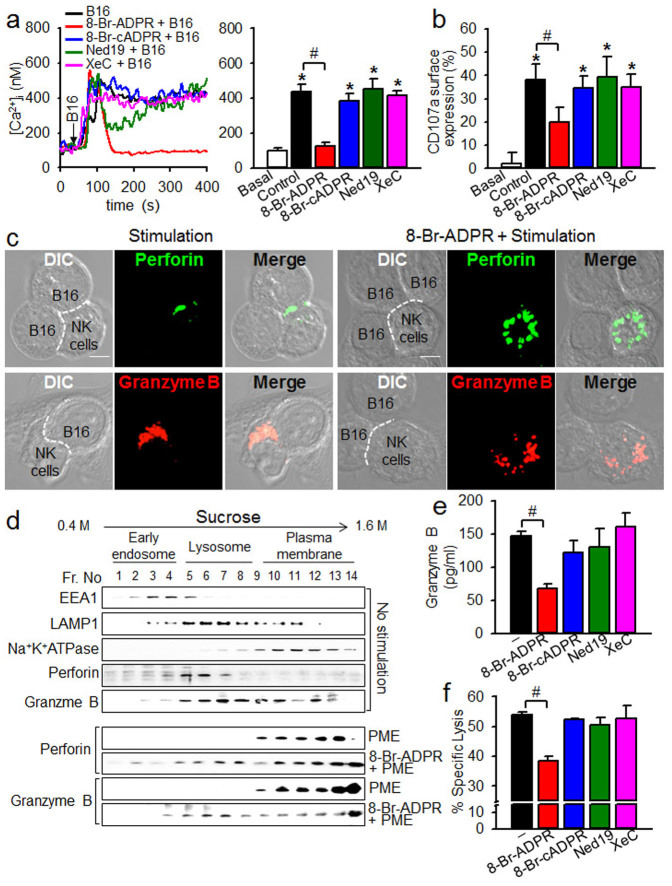
ADPR-mediated Ca^2+^ signals are required for cytolytic degranulation of NK cells. (a) 8-Br-ADPR inhibits tumor cell-induced sustained Ca^2+^ signals in NK cells. NK cells were pretreated with 100 μM 8-Br-ADPR, 100 μM 8-Br-cADPR, 10 μM Ned19, or 2 μM XeC for 20 min. Arrow indicates the time of addition of B16F10 cells. (b) Inhibition of tumor cell-induced NK cell degranulation by 8-Br-ADPR. Data shown in a and b are representative of three independent experiments. *P < 0.001 vs basal; ^#^*P* < 0.05. (c) 8-Br-ADPR inhibits tumor cell-induced translocation of perforin and granzyme B towards immunological synapses. NK cells were added to B16F10 cells at 1:1 ratio at 37°C for 20 min and stained with perforin or granzyme B. The dashed line indicates the immunological synapse. (Scale bars, 5 μm). All images were representative of at least three independent experiments. (d) NK cells were treated with PME (30 μg) for 20 min, and homogenized and fractionated on a continuous 0.4 M to 1.6 M sucrose density gradient using ultracentrifugation. Fractions were analyzed by immunoblotting to identify cellular organelles and identified using antibodies against each marker: early endosome (EEA1), lysosomes (LAMP1), plasma membrane (Na^+^K^+^-ATPase). Distributions of granzyme B and perforin were assessed by immunoblotting. Shown is a representative of three independent experiments. The gels have been run under the same experimental conditions. The full-length blots and the cropped blots are presented in Supplementary Figure 2. (e and f) 8-Br-cADPR inhibits tumor-induced granzyme B secretion (e) and cytotoxicity activity (f) of NK cells. 100 μM 8-Br-ADPR, 100 μM 8-Br-cADPR, 10 μM Ned19, or 2 μM XeC was preincubated for 20 min. Data are mean ± SEM of three independent experiments. ^#^*P* < 0.001.

We examined whether various Ca^2+^ signaling messengers produced by CD38 were actually involved in tumor cell-induced Ca^2+^ signals and degranulation in NK cells. To this end, we tested various inhibitors of the CD38 signaling pathway to observe their effects on tumor-induced degranulation and Ca^2+^ signals as shown in Fig. 2. Among the inhibitors that we tested (8-Br-ADPR, an antagonistic analog of ADPR; 8-Br-cADPR, a cADPR antagonist; Ned19, an NAADP antagonist; and Xestospongin C (XeC), an IP_3_ receptor antagonist), only 8-Br-ADPR blocked the late, sustained portion of tumor-induced Ca^2+^ signals (Fig. 3a) and degranulation (Fig. 3b). The effects of 8-Br-ADPR were confirmed by confocal microscopic findings that 8-Br-ADPR blocked the tumor cell-induced translocation of perforin and granzyme B towards the immunological synapse between B16F10 and NK cells (Fig. 3c).

To confirm the inhibitory effects of 8-Br-ADPR on the translocation of intracellular perforin and granzyme B to the plasma membrane in response to tumor cell contact, we examined the subcellular distributions of perforin and granzyme B by Western blot. Subcellular organelles of NK cells were separated by sucrose density gradient centrifugation. Perforin and granzyme B were enriched in higher density fractions, consisting of plasma membrane from NK cells treated with plasma membrane extracts (PME) from B16F10 cells, compared to those from NK cells that had not been treated (Fig. 3d). 8-Br-ADPR inhibited the tumor-induced translocation of cytoplasmic granular proteins to the plasma membrane. These results are consistent with those in Fig. 3c, showing that intracellular perforin and granzyme B were translocated to the plasma membrane in response to tumor contact. Furthermore, we found that the B16F10 cell-induced granzyme B release from NK cells, as well as the cytolytic activity of NK cells against B16F10 cells, were inhibited by 8-Br-ADPR but not by any other inhibitors we tested (Fig. 3e and f). Taken together, these results suggest that ADPR is a key player in mediating the degranulation and cytolytic activity of NK cells.

### Intracellular production of ADPR is necessary for the cytolytic activity of NK cells against tumors and depends on CD38 signaling, which facilitates sustained Ca^2+^ signals

**Figure 4:**
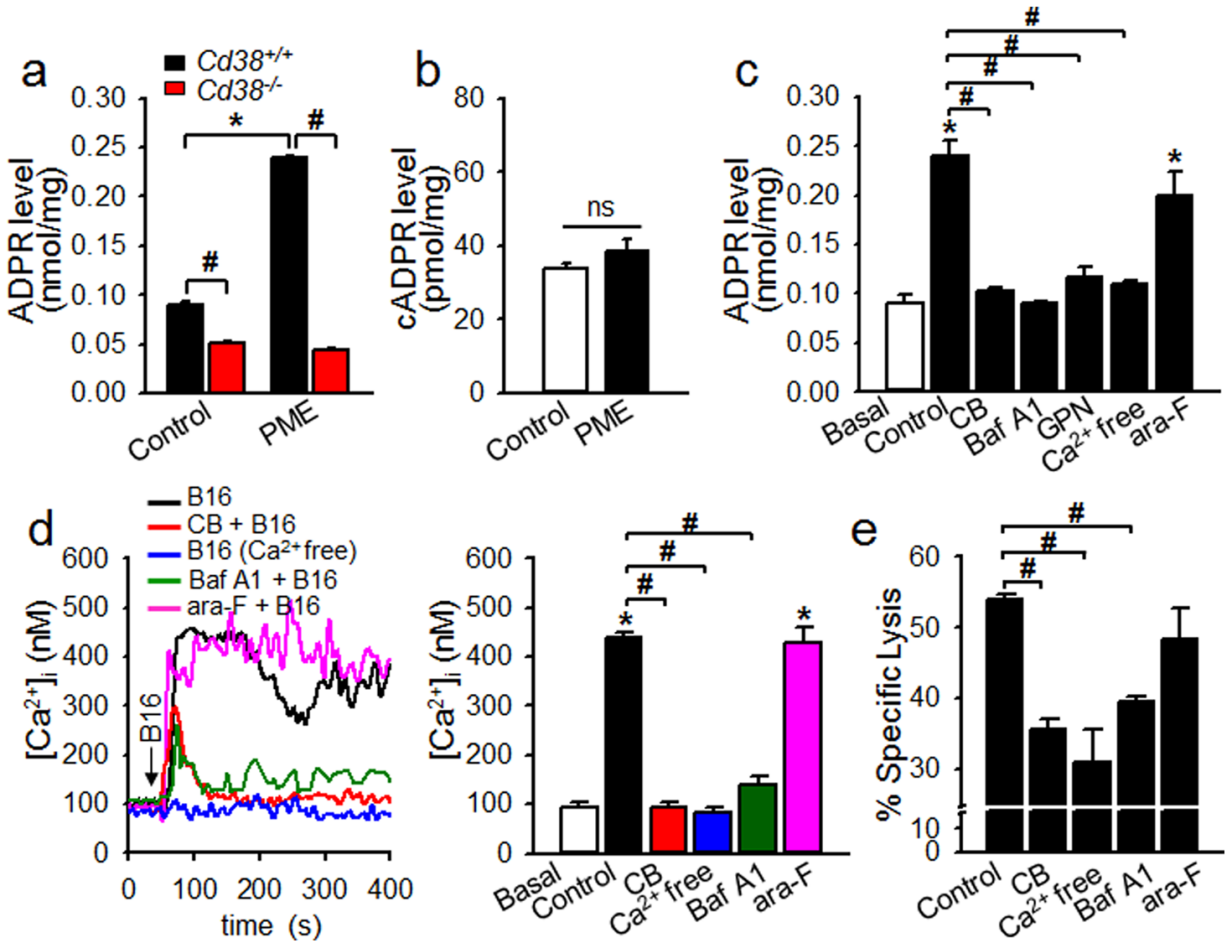
Tumor-induced ADPR production by intracellular CD38 is responsible for sustained Ca^2+^ signals in NK cells. (a and b) Tumor cells induce ADPR production but cADPR in *Cd38^+/+^* NK cells. Levels of ADPR and cADPR were determined after treatment of NK cells with 30 μg PME prepared from B16F10 tumor cells for 40 s. Data are mean ± SEM of three independent experiments. *P < 0.001; ^#^*P* < 0.05. ns, not significant. (c–e) Cibacron blue 3GA (CB) inhibits of tumor-stimulated ADPR induction (c), sustained Ca^2+^ signal (d), cytotoxicity (e). CB (100 μM), ara-2′-F-NAD (200 nM), bafilomycin A1 (200 nM) or GPN (50 μM) was preincubated for 30 min. Data shown in c–e are representative of three independent experiments. *P < 0.001 vs basal; ^#^*P* < 0.05.

Because 8-Br-ADPR, an antagonistic analog of ADPR, inhibited the degranulation and cytolytic activity of NK cells, we investigated whether ADPR was endogenously produced in tumor cell-stimulated NK cells and if CD38 was the enzyme responsible for ADPR production. The level of ADPR in *Cd38^+/+^* NK cells increased 2.4 fold when stimulated by PME, which was not observed in *Cd38^−/−^* NK cells (Fig. 4a). By contrast, the level of cADPR, another Ca^2+^ signaling second messenger produced by CD38, was not affected by stimulation with the PME of tumor cells (Fig. 4b). These results show that ADPR is specifically induced by CD38 in response to tumor cells.

CD38 is primarily located at two sites, the plasma membrane and intracellular organelles^28^,^29^,^30^,^31^, Therefore, we investigated the involvement of intracellular and extracellular CD38 in inducing ADPR by testing two CD38 inhibitors^13^,^32^, Cibacron blue 3GA (a cell-permeable CD38 inhibitor) and ara-2′-F-NAD (a cell-impermeable CD38 inhibitor). The former inhibited tumor cell-stimulated ADPR production, sustained Ca^2+^ increase, and cytotoxicity, whereas the latter did not (Fig. 4c–e), indicating that CD38 in intracellular organelles is responsible for the ADPR production in response to tumor PME stimulation. Interestingly, lysosomal disrupting reagents such as bafilomycin A1 (Baf A1), an inhibitor of vacuolar-type H^+^-ATPase that prevents the re-acidification of acidic organelles, or glycyl-phenylalanine-2-naphthylamide (GPN), a reagent that osmotically ruptures lysosomes, also inhibited NK cells from inducing ADPR production in response to tumor PME stimulation (Fig. 4c).

Tumor-induced ADPR production was abolished in media devoid of extracellular Ca^2+^, suggesting that the initial transient Ca^2+^ influx was a central upstream pathway for the induction of ADPR. Cibacron blue 3GA, Baf A1, as well as extracellular Ca^2+^-free conditions, which prevent NK cells from inducing ADPR production and maintaining an elevated [Ca^2+^]_i_ in response to tumor cells, diminished the cytolytic activity of NK cells against B16F10 cells (Fig. 4c–e). This suggests that tumor cell-induced production of ADPR, which is necessary for the cytolytic activity of NK cells, depends on the initial transient Ca^2+^ influx, followed by sustained Ca^2+^ concentration achieved by CD38 signaling.

### Tumor-induced ADPR production depends on PKA activation

**Figure 5:**
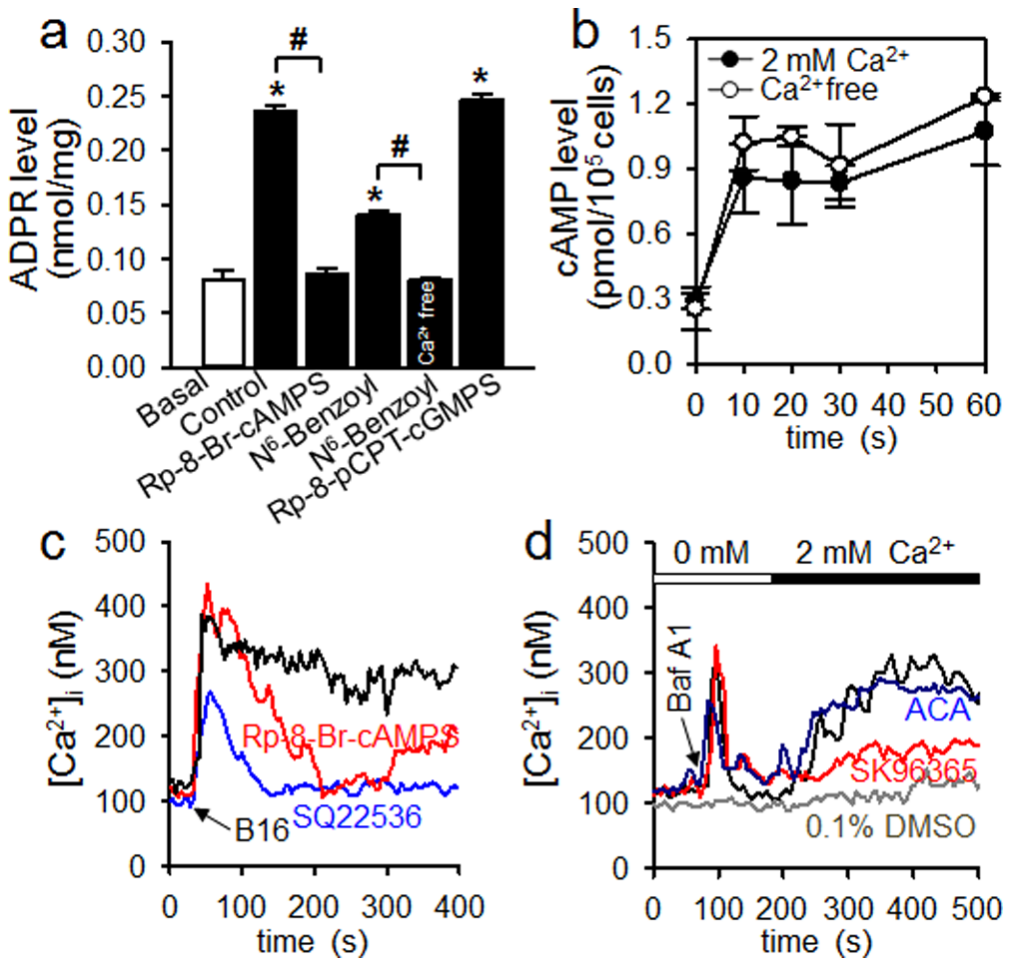
Tumor cells produce ADPR by activation of PKA but not PKG in NK cells. (a) ADPR levels were determined after treatment of NK cells with 30 μg PME for 40 s. Rp-8-Br-cAMPS (100 μM) or Rp-8-pCPT-cGMPS (20 μM) was preincubated for 30 min. 100 μM *N*^6^-benzoyl-cAMP (PKA activator, 40 s) was used for ADPR measurement. Data are mean ± SEM of three independent experiments. *P < 0.001 vs basal; ^#^*P* < 0.05. (b) cAMP levels were determined after treatment of NK cells with 30 μg PME. Data are mean ± SEM of three independent experiments. (c) Inhibition of tumor cell-induced sustained Ca^2+^ increase by a PKA inhibitor, Rp-8-Br-cAMPS, and an adenylate cyclase inhibitor, SQ 22536. Rp-8-Br-cAMPS (100 μM) or SQ 22536 (250 μM) was preincubated for 30 min. (d) SOCE induced by bafilomycin A1. Bafilomycin A1 (1 μM)-induced SOCE was inhibited with 50 μM SK96365 but not by 20 μM ACA. SK96365 or ACA was pre-incubated for 30 min. Data shown in c and d are mean ± SEM of three independent experiments. n = 10.

It has been demonstrated that ADPR-cyclases, including CD38, are activated by PKA or PKG^29^,^33^,^34^,^35^. To elucidate the upstream signaling pathways of ADPR production by CD38 in tumor-induced NK cell activation, we determined [ADPR]_i_ in NK cells following treatments with various agonistic and antagonistic agents. Tumor cell-induced ADPR production was inhibited by Rp-8-Br-cAMPS (a membrane-permeable PKA inhibitor), but not Rp-8-pCPT-cGMPS (a membrane-permeable PKG inhibitor). Moreover, N^6^-Benzoyl (a membrane-permeable PKA activator) significantly increased [ADPR]_i_, but this increase was inhibited in Ca^2+^-free conditions (Fig. 5a). These findings suggest that the initial transient Ca^2+^ influx and cAMP/PKA are required for CD38 activation in tumor-induced ADPR production. We further examined whether cAMP was produced in tumor cell-stimulated NK cells. cAMP was produced after treating NK cells with PME, regardless of extracellular Ca^2+^ levels (Fig. 5b), suggesting that tumor-induced cAMP production is independent of initial transient Ca^2+^ influx. Furthermore, the tumor cell-induced sustained [Ca^2+^]_i_ increase was inhibited by Rp-8-Br-cAMPS or SQ 22536 (an adenylyl cyclase inhibitor) (Fig. 5c). These findings suggest that both the initial Ca^2+^ influx and cAMP/PKA are required in order for CD38 activation to produce ADPR in tumor-induced NK cells.

To test the contribution of store-operated Ca^2+^ entry (SOCE) to the tumor cell–induced sustained Ca^2+^ signal, we used Baf A1 to deplete Ca^2+^ from acidic organelles, which are the Ca^2+^ stores responsible for tumor cell-mediated Ca^2+^ signals stimulated by ADPR (Fig. 4c and d). Depletion of Ca^2+^ stores by Baf A1 induced Ca^2+^ entry after Ca^2+^ was added to the bath perfusion solution, as described earlier^36^ (Fig. 5d). This SOCE was completely blocked by SK96365, a SOCE blocker, but not by ACA, a specific TRPM2 channel blocker. These findings indicate that the sustained Ca^2+^ signal is attributable to SOCE following Ca^2+^ mobilization by ADPR, and that TRPM2 is not involved in ADPR-mediated SOCE.

### TRPM2 is required for the migration of perforin-containing granules towards the immunological synapse as NK cells encounter tumor cells

**Figure 6:**
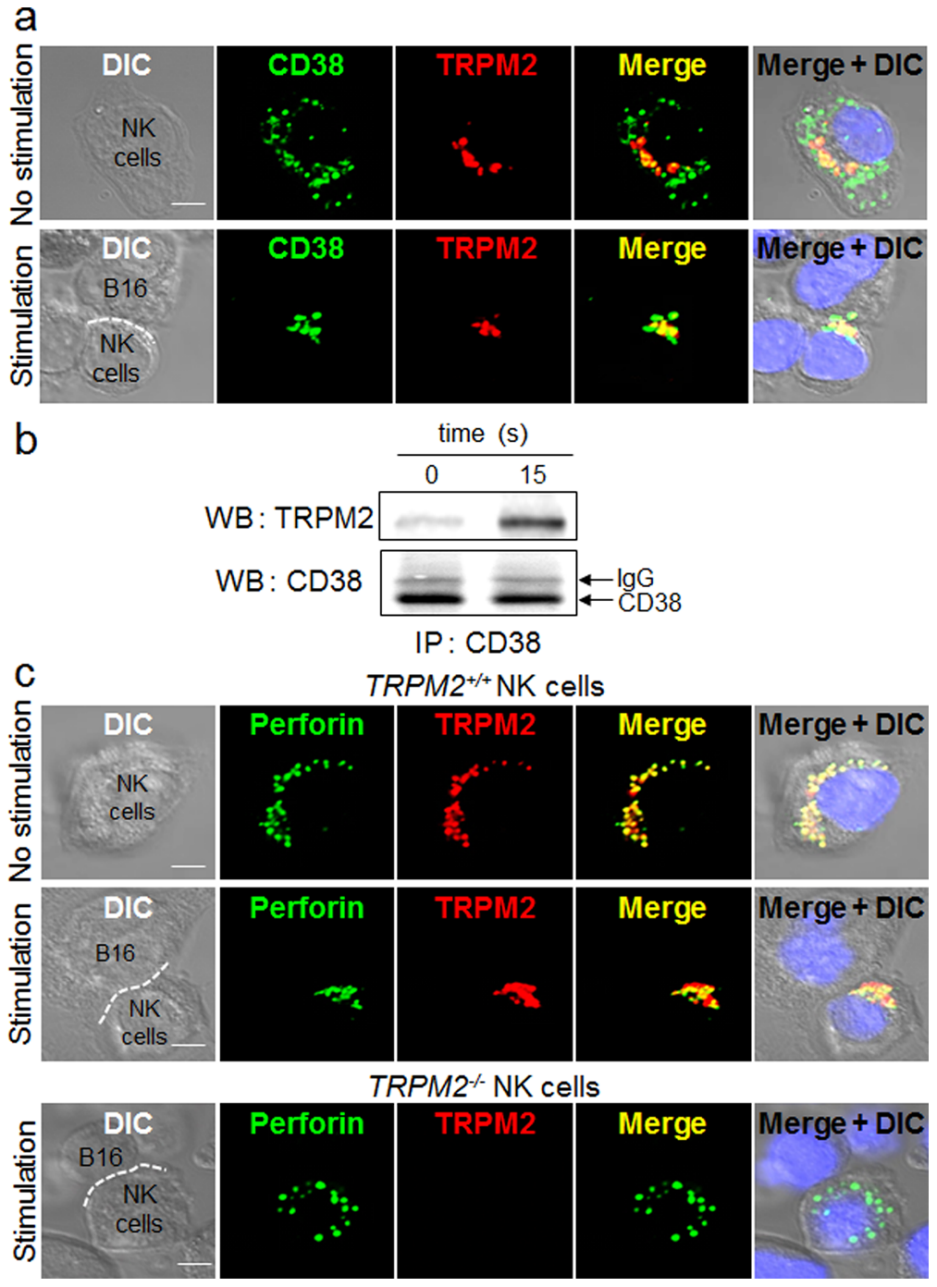
Tumor cell increases the interaction of CD38 with TRPM2 in NK cells. (a) Increased co-localization of CD38 with TRPM2 in NK cells upon tumor cell treatment. NK cells were incubated with B16F10 cells for 20 min and then stained with CD38 and TRPM2. (b) Tumor cell PME increases association of CD38 with TRPM2 in NK cells. NK cells were treated with PME for 15 s. Cells were extracted with a lysis buffer and then subjected to immunoprecipitation (IP) using an anti-CD38 mAb. Immunoprecipitated proteins were analyzed by western blotting (WB) with anti-CD38 pAb or anti-TRPM2 pAb. The gels have been run under the same experimental conditions. The full-length blots and the cropped blot are presented in Supplementary Figure 3. (c) Defect of tumor cell-stimulated translocation of perforin towards immunological synapses in *TRPM2^−/−^* NK cells. *TRPM2^+/+^* or *TRPM2^−/−^* NK cells were added to B16F10 target cells at 1:1 ratio at 37°C for 20 min and then stained with perforin and TRPM2. DAPI was used as a nuclear stain (blue). The dashed line indicates the immunological synapse. (Scale bar, 5 μm). All images were representative of at least three independent experiments.

Because ADPR is known to activate TRPM2 in order to elicit Ca^2+^ release from intracellular stores^15^,^16^, we focused on studying TRPM2 to explore its possible role in perforin exocytosis. To this end, we first examined whether CD38 co-localized with TRPM2 in NK cells. CD38 and TRPM2 were dispersed at different locations in non-stimulated NK cells. However, contact with B16F10 cells changed the locations of CD38 and TRPM2, concentrating them at the immunological synapse formed between NK cells and B16F10 cells (Fig. 6a). Furthermore, CD38 and TRPM2 proteins appeared to physically interact only after NK cells were stimulated by B16F10 cells, as judged by the co-immunoprecipitation of TRPM2 with CD38 (Fig. 6b). These results suggest that the formation of ADPR by CD38 in NK cells leads to activation of TRPM2 channels, due to the proximity of TRPM2 and CD38.

Similar to the findings observed with CD38 and TRPM2 in NK cells, perforin was scattered inside the NK cells mostly inside cytolytic granules and co-localizing with TRPM2 even before stimulation with tumor cells (Fig. 6c, upper panel). Like CD38, perforin co-migrated with TRPM2 to the immunological synapse upon stimulation with B16F10 cells (Fig. 6c, middle panel). Such tumor cell-induced perforin migration was not detected in *TRPM2^−/−^* NK cells (Fig. 6c, lower panel), suggesting that TRPM2 was essential for perforin migration towards the immunological synapse. The finding that perforin migration towards the synapse requires CD38 as well (Fig. 2b) suggests that both CD38 and TRPM2 are absolutely essential for the migration of perforin (or possibly cytolytic granules as well) toward the immunological synapse when NK cells encounter tumor cells.

### Adoptive transfer of *Cd38^+/+^* NK cells, but not *Cd38^−/−^* NK cells, controls B16F10 melanoma lung metastasis

**Figure 7:**
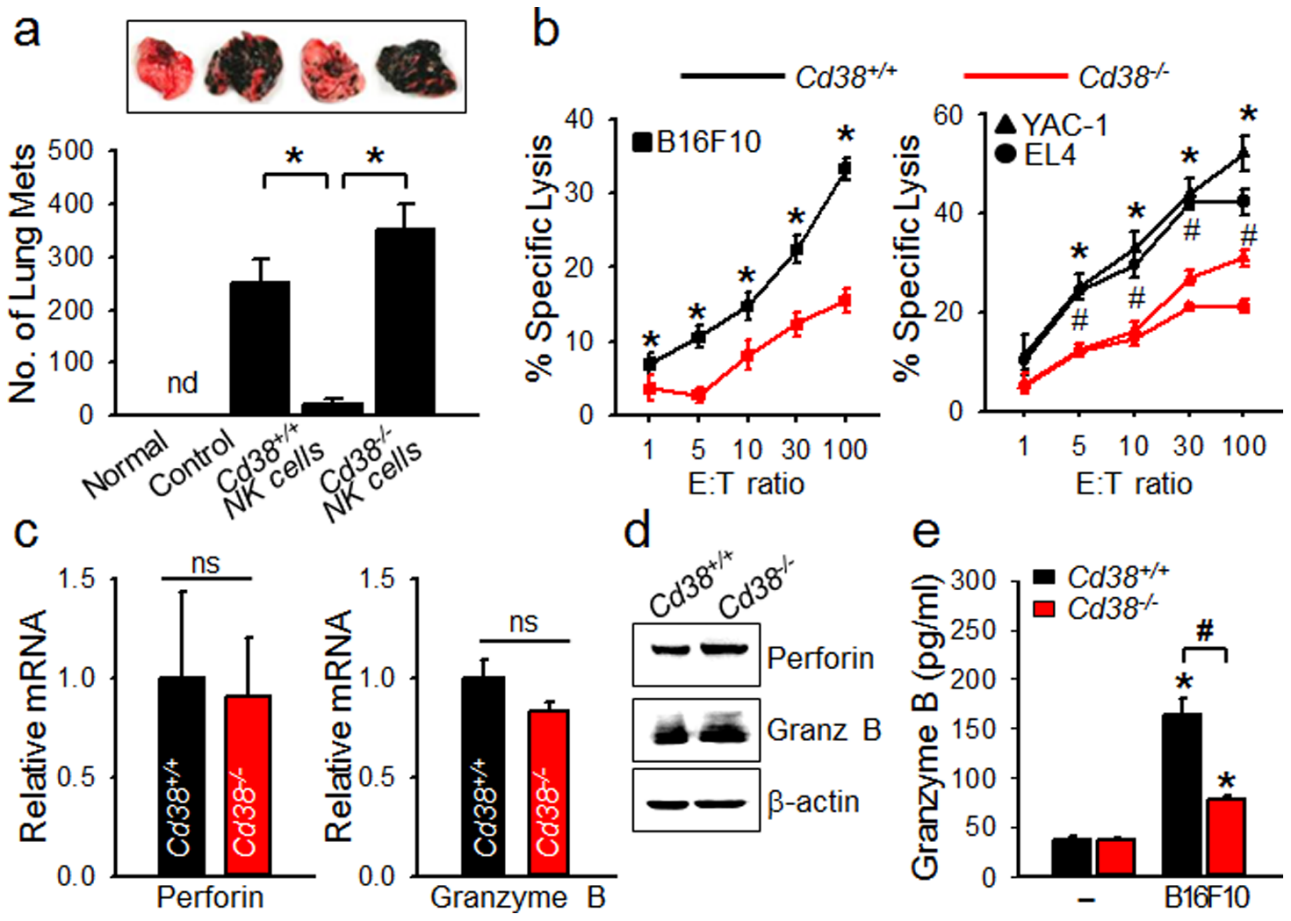
CD38 is critical for the antitumor effects of NK cells via cytolytic granule exocytosis. (a) Antitumor effects of *Cd38^+/+^* and *Cd38^−/−^* NK cells in a lung metastasis model of B16F10 cells. C57BL/6 mice (n = 4 per cohort) were injected i.v. with 1 × 10^5^ B16F10 melanoma cells. After 2 d, IL-2 activated NK cells (2 × 10^6^ cells) from *Cd38^+/+^* or *Cd38^−/−^* mice were injected i.v. into B16F10-bearing wild-type mice. Lungs were harvested 14 d later and metastatic nodules were counted. nd, not detected. Data are mean ± SEM of three independent experiments. Representative images of lungs are shown. *P < 0.001. (b) Comparison of cytolytic activity of *Cd38^+/+^* and *Cd38^−/−^* NK cells against various target cell lines. *Cd38^+/+^* or *Cd38^−/−^* NK cells cultured for 10 d were assessed for cytolytic activity as indicated, over 4 h. Data are mean ± SEM of three independent experiments. *P < 0.001; ^#^*P* < 0.05. (c and d) *Cd38^+/+^* and *Cd38^−/−^* NK cells are expressed equivalent amounts of perforin and granzyme B at mRNA (c) and protein levels (d). The gels have been run under the same experimental conditions. The full-length blots and the cropped blots are presented in Supplementary Figure 4. Data are mean ± SEM of three independent experiments. ns, not significant. (e) Comparison of the amount of granzyme B released into the media by *Cd38^+/+^* and *Cd38^−/−^* NK cells on stimulation with B16F10 cells for 30 min. Data are mean ± SEM of three independent experiments. *P < 0.001 vs basal; ^#^*P* < 0.05.

Based on the above findings, we extended our study to the B16F10 experimental metastasis model in C57BL/6 mouse to test whether the adoptive transfer of NK cells to mice with B16F10 cell metastasis could reduce the number of tumor nodules and increase survival rates. Indeed, *Cd38^+/+^* NK cells significantly reduced the number of metastasized B16F10 cells in the lung, which did not hold true for *Cd38^−/−^* NK cells, indicating that CD38 was essential for the antitumor activity of NK cells (Fig. 7a). *In vitro* cytotoxic assays demonstrated that the cytolytic activity of *Cd38^+/+^* NK cells was superior to that of *Cd38^−/−^* NK cells against not only B16F10 but also YAC-1 and EL4 murine lymphoma cell lines (Fig. 7b), indicating that *Cd38^+/+^* NK cells exhibited a broad spectrum of natural cytotoxicity. Both *Cd38^+/+^* and *Cd38^−/−^* NK cells expressed equivalent amounts of perforin and granzyme B mRNA and protein levels (Fig. 7c and d). However, B16F10-induced secretion of granzyme B was lower in CD38^−/−^ NK cells than CD38^++^ NK cells (Fig. 7e). These results suggest that the enhancing effect of CD38 on the cytolytic activity of NK cells results from the stimulated release of granzyme B to target cells.

## Discussion

The cytolytic activity of NK cells requires an initial Ca^2+^ influx for conjugation with the tumor cells, and intracellular Ca^2+^ mobilization is required for granule polarization and the degranulation process^7^. However, the activation processes that lead to the polarized degranulation of NK cells are not well understood. We have demonstrated here that cytolytic granule exocytosis and the antitumor functions of NK cells rely upon signaling pathways involving CD38, ADPR, and TRPM2, and Ca^2+^ release from acidic stores. The inhibition of this signaling pathway significantly decreased the cytolytic granule exocytosis and antitumor activity in both in vitro and in vivo models.

The role of CD38 as an adhesion molecule in NK cell antitumor function has been demonstrated in earlier studies^26^. However, the proposed function of CD38 as an adhesion molecule is not likely, as evidenced by the lack of difference in the formation of conjugates between the tumor and *Cd38^+/+^* and *Cd38^−/−^* NK cells (Fig. 2a). Our data reveal that the production of ADPR by CD38 in response to tumor cells was ablated in *Cd38^−/−^* NK cells (Fig. 4a), indicating that CD38 was responsible for the production of ADPR in response to tumor cells. Intriguingly, cADPR has been known as a Ca^2+^ signaling second messenger involved in IL8-induced chemotaxis in lymphokine activate killer cells^35^. Our data shown in Fig. 4b, which showed no significant increase of cADPR in tumor stimulated NK cells, rule out the possibility that ADPR is produced from cADPR, another one of CD38's products. This indicates that the interaction with tumor cells through cognate receptors on NK cells transmits signals for the activation of CD38's NAD glycohydrolase (NADase) to produce ADPR. Further studies will be required to identify the mechanisms regarding how specific enzymatic activities of CD38 are selectively activated under specific situations.

We found that the sustained Ca^2+^ signaling, mediated by ADPR and TRPM2, was correlated with polarized degranulation and cytolytic activity of NK cells against the melanoma tumor cell line B16F10. Furthermore, we have shown the importance of acidic environments for CD38 activity in NK cells, because the presence vacuolar H^+^ pump inhibitors resulted in the failure of ADPR production, Ca^2+^ mobilization, and cytolytic activity induced by CD38. All the CD38 activities in NK cells we described depend exclusively on the tumors contacted or the PME prepared from tumor cells. Finally, our results unveiled the critical role of TRPM2 in NK cell cytotoxicity against tumor cells when NK cells were introduced to mice bearing B16F10 cells as passive immunotherapy.

TRPM2 is expressed in the plasma membranes as well as in intracellular compartments. The functions of intracellular TRPM2 have been studied only on insulin secretion in pancreatic β cells^15^ and the maturation and chemotaxis of dendritic cells^16^. We show that TRPM2 is an ion channel located in the cytolytic granule, and co-migrates with CD38 to the immunological synapse upon tumor stimulation (Fig. 6). Like CD38, TRPM2 appears to be essential for sustained Ca^2+^ signals, directed degranulation, and cytolytic activity, suggesting that CD38 is tightly coupled to TRPM2 ion channel in inducing sustained Ca^2+^ signals, which may be pre-requisite for the directed migration of cytolytic granules to the immunological synapses. Therefore, cytolytic granules contain all the necessary machinery for Ca^2+^ signaling, including Ca^2+^ stores and exocytosis capabilities. The lack of either CD38 or TRPM2 results in a significant increase of tumor formation and reduced survival rates. Thus, CD38 and TRPM2 cooperate to generate the sustained Ca^2+^ signal and directed migration of cytolytic granules to the immunological synapse in response to tumor stimulation.

Our data show that the majority of TRPM2 is not expressed in the plasma membrane, but is localized in intracellular granules (Fig. 6). Although our data (Fig. 5d) show that TRPM2 is not involved in SOCE, we cannot rule out a possibility that the granular TRPM2 can gain access to external Ca^2+^ only after degranulation and fusion of the granules with the plasma membrane. To clarify the cause-effect relationship between translocation of TRPM2 and Ca^2+^ influx, we compared the time course of the tumor cell-induced translocation of TRPM2 and Ca^2+^ influx at 5 min after stimulation with B16F10 cells and found that TRPM2 remained at intracellular granules (Supplementary Figure S1) when the tumor cell-induced Ca^2+^ influx occurred (Fig. 1a and 2e). These findings suggest that Ca^2+^ influx precedes degranulation, and that the sustained Ca^2+^ signal is required for degranulation. Taken together, our data indicate that an ion channel other than TRPM2 is responsible for ADPR-mediated SOCE.

In CTLs, TCR activation recruits NAADP to activate TPC channels present on cytolytic granules^9^. These cytolytic granules store and release Ca^2+^, although SOCE through the Orai1-STIM1 complex is necessary for lytic granule exocytosis and tumor cell killing^8^. Our data show that NAADP is not involved in tumor-induced cytolytic degranulation in NK cells (Fig. 3). Thus, different cells use different Ca^2+^ signaling messengers for similar cellular events, and such interesting mechanisms remain to be determined.

As described, polarized cytolytic degranulation towards the immunological synapse is likely guided by specific Ca^2+^ channels. Our data affirm a key role of the CD38/ADPR/TRPM2 pathway in generating polarized cytolytic degranulation towards the immunological synapses via Ca^2+^ signals from cytolytic granules that subsequently lead to target cell killing. We found that the sustained Ca^2+^ signaling was a pre-requisite for polarized degranulation and lysis of the target cell in vitro. TRPM2 serves to couple CD38/ADPR activation with sustained Ca^2+^ signals and contributes to potentiating NK cell cytotoxicity against tumor cells in the mouse model. Understanding how suitable and specific Ca^2+^ mobilization signals drive the polarized migration of cytolytic proteins towards the immunological synapse to kill target cells is important. This study highlights the selective role of CD38 in stimulating directional exocytosis, which is crucial for target cell killing and may help to design better immunotherapy through the use of NK cells.

In conclusion, we demonstrate that ADPR is produced by CD38, localized in cytolytic granules, upon tumor stimulation. ADPR modulates Ca^2+^ signaling by gating TRPM2 channels, and causes cytolytic polarized degranulation from NK cells. These data constitute a novel mechanism of Ca^2+^ signaling for the antitumor functions of NK cells, and may provide an antitumor therapeutic strategy utilizing increased NK cell cytotoxicity.

## Methods

### Reagents

Antibodies were obtained as follows: granzyme B pAb from Cell Signaling (Danvers, MA) and perforin pAb from Santa Cruz Biotechnology (Santa Cruz, CA); anti-perforin mAb, DyLight 488 anti-perforin mAb, and DyLight 550 anti-TRPM2 pAb from Novus Biologicals (Littleton, CO). Human recombinant IL-2 was from Chiron BV (Amsterdam, Netherlands) and Xestospongin C was from Santa Cruz Biotechnology (Santa Cruz, CA). 8-Br-ADPR, ara-2′-F-NAD, and *N*-(*p*-amylcinnamoyl) anthranilic acid (ACA) were from Biolog Life Science Institute (Bremen, Germany). All other reagents were obtained from Sigma-Aldrich (St. Louis, MO).

### Mice

*Cd38*^−/−^ mice (B6.129P2-Cd38^tm/Lud^) were purchased from Jackson Laboratory (Bar Harbor, ME). TRPM2^−/−^ mice were kindly provided by Mori (Kyoto University, Japan). Mice were bred and housed in the facilities of Chonbuk National University Medical School under specific pathogen free conditions. All experimental animals were used under a protocol approved by the institutional animal care and user committee of the Chonbuk National University Medical School (CBU 2014-00031). Standard guidelines for laboratory animal care were performed in accordance with the Guide for the care and use of laboratory animals published by the National Institutes of Health^37^.

### Cell culture

Mouse NK cells were isolated by negative immunomagnetic selection (Stem cell Technologies). The cells were cultured in a 5% CO_2_ incubator at 37°C in culture media containing 5000 IU/ml IL-2 for 10 d. The culture medium used was RPMI-1640 supplemented with 10% FBS, 0.25 μg/ml amphotericin B, 10 U/ml penicillin G, 100 μg/ml streptomycin, 1 mM L-glutamine, 1% nonessential amino acids (Invitrogen), and 50 μM 2-mercaptoethanol. Purity of NK cells was determined by FACS analysis. The murine melanoma B16F10 and lymphoma EL4 and YAC-1 cells were purchased from ATCC (Manassas, VA).

### Animal model

To establish tumor in the flanks of C57BL/6 and *TRPM2^−/−^* mice, tumor cells were resuspended in 200 μl of PBS and injected subcutaneously. The tumor developed at the site of injection was measured and the mean tumor size was calculated [length (cm) × width (cm)]. To produce experimental lung metastasis, C57BL/6 mice received intravenous injections of B16F10 cells (1 × 10^5^ cells/mouse). After 2 d, NK cells (2 × 10^6^ cells/mouse) from *Cd38^+/+^*, *Cd38^−/−^*, or *TRPM2^−/−^* mice were injected intravenously into wild-type or *TRPM2^−/−^* mice. Mice were sacrificed 14 d later. Lungs were fixed with 4% paraformaldehyde and metastatic nodules on the surface of the lungs were counted.

### Calcium imaging

NK cells were loaded with 5 μM Fluo-4 AM (Invitrogen) in HBSS at 37°C for 40 min and changes in [Ca^2+^]_i_ were determined at 488 nm excitation/530 nm emission wavelengths using a confocal microscope (Nikon). For calculation of [Ca^2+^]_i_, the method of Tsien *et al.*^38^ was used with the following equation: [Ca^2+^]_i_ = *K_d_*(*F* − *F*_min_)/(*F*_max_ − *F*), where *K_d_* is 335 nM for Fluo-4, and *F* is the observed fluorescence. Each tracing was calibrated for maximal intensity (*F*_max_) by addition of ionomycin (8 μM) and for minimal intensity (*F*_min_) by addition of EGTA 50 mM after each measurement.

### Measurement of [ADPR]_i_

ADPR level was measured using LC-MS/MS as described previously^39^. Briefly, cells were treated with 5% trichloroacetic acid under sonication, and precipitates were removed by centrifugation at 20,000 × *g* for 10 min. Supernatants were loaded onto a Waters ACQUITY UPLC system coupled to a Waters Xevo TQ-S mass spectrometer and separated using a BEH Amide column (Waters ACQUITY UPLC BEH Amide, 130 Å, 1.7 μm, 2.1 mm × 50 mm). The column was equilibrated with 100% buffer B (90% acetonitrile/10% 50 mM ammonium formate), and eluted with a 5-min gradient to 60% buffer A (10 mM ammonium formate in water) at a flow rate of 0.5 ml/min. The following parameters were used for ADPR MS analysis: cone gas, 150 l/h; nebulizer, 7 Bar; and desolvation temperature, 350°C. Ion transitions used for confirmation and quantification of ADPR were m/z 558.17→346.01.

### Measurement of [cADPR]_i_

cADPR levels were measured with a previously described cycling method^40^. Increases in resorufin fluorescence were measured at 544 nm excitation/590 nm emission wavelengths using a SpectraMax Gemini fluorescence plate reader (Molecular Devices Corp.).

### Measurement of [cAMP]_i_

cAMP levels were determined by a cAMP immunoassay kit according to the manufacturer's protocol (Enzo Life Science). NK cells were preincubated with 0.5 mM isobutylmethylxanthine, a phosphodiesterase inhibitor. After incubation with PME for the indicated times, the cells were lysed in 0.1 M HCl to stop the reaction. After centrifugation at 20,000 × *g* for 10 min at 4°C, supernatants were collected and acetylated, and immunoassays were performed.

### Quantitative RT-PCR

Total RNA was isolated from NK cells using an RNeasy Mini Kit (Qiagen, Valencia, CA). cDNA was synthesized by reverse transcription from 50 ng total RNA using a cDNA Reverse Transcriptase Kit (TaKaRa, Japan). The PCR reaction was carried out in 384-well plate using the ABI Prism 7900HT Sequence Detection System (Applied Biosystems). Real-time PCR primers for perforin, granzyme B, and GAPDH were as follows: perforin (forward, 5′-AGCACAAGTTCGTGCCAGG-3′, and reverse, 5′-GCGTCTCTCATTAGGGAGTTTTT-3′); granzyme B (forward, 5′-CCACTCTCGACCCTACATGG-3′, and reverse, 5′-GGCCCCCAAAGTGACATTTATT-3′); GAPDH (forward, 5′-CATGGCCTTCCGTGTTCCTA-3′, and reverse, 5′-ATGCCTGCTTCACCACCTTCT-3′).

### Formation of conjugation

NK cells were stained with PKH-26 (red), and B16F10 cells were stained with CellTrace CFSE (green) according to the manufacturer's instructions. Effector and target cells (E/T ratio: 1/1) were co-cultured at 37°C. At the time point, the samples were fixed with ice-cold 0.5% paraformaldehyde. The double-positive population was assessed using flow cytometry (FACSAria).

### Degranulation assay

NK cell degranulation was assessed as previously described^41^. Briefly, 2 × 10^5^ NK cells were added to 2 × 10^5^ target cells in 200 μl of complete medium. Cells were mixed, centrifuged for 3 min at 20 × *g*, and incubated for 2 h at 37°C. Thereafter, cells were centrifuged, stained with fluorochrome-conjugated mAbs against NK1.1 and CD107a (BD Biosciences) in PBS supplemented with 2% FBS and 2 mM EDTA for 45 min on ice, washed, re-suspended in PBS supplemented with 2% FBS and 2 mM EDTA, and analyzed by flow cytometry. Data were analyzed with FlowJo version10 software.

### Cytokine measurement

NK cells were added to 4 × 10^5^ B16F10 cells in 200 μl complete medium. Cells were incubated for 30 min at 37°C in a 5% CO_2_ incubator. Thereafter, supernatants were collected and cytokines were assayed with ELIZA kits from eBioscience (granzyme B) according to the instructions of the manufacturer.

### Cytotoxicity

NK cell cytotoxicity was assessed as previously described^42^. Target (T) cells labeled with 5 μM calcein AM (Invitrogen) for 1 h at 37°C and effector (E) cells were co-incubated with 200 μl complete medium in a 96 well plate for 4 h at 37°C. Spontaneous release of calcein was determined by incubating loaded target cells in medium alone, and maximal release was determined by adding 0.1% Triton X-100 to lyse all target cells. After incubation, plates were centrifuged at 300 × *g* for 5 min, and 100 μl of supernatant from each sample was transferred to a 96-well plate. Fluorescence was measured at 480 nm excitation/538 nm emission wavelengths. Cytotoxicity, measured as a percent specific release of calcein, was calculated using the following formula: percent specific release = (experimental release - spontaneous release)/(maximum release - spontaneous release) × 100.

### Immunoprecipitation and immunoblotting

Immunoprecipitation and immunoblotting were carried out as described previously^29^.

### Plasma membrane preparation and subcellular fractionation

For preparation of B16F10 cell plasma membranes, cells were homogenized with a Dounce homogenizer in 0.5 ml of homogenate buffer (0.25 M sucrose, 0.5 mM EDTA, 10 mM HEPES, pH 7.4 and 1 mM PMSF). The homogenate was centrifuged at 1000 × *g* for 10 min at 4°C, supernatants were centrifuged at 20,000 × *g* for 20 min at 4°C, and then supernatants were ultracentrifuged at 100,000 × *g* for 2 h at 4°C. The pellet was washed with ice-cold PBS and then used as plasma membrane. NK cells were homogenized as above and the homogenate was subcellular fractionated as described previously^29^.

### Confocal imaging

NK cells were mixed with B16F10 cells at 1:1 ratio at 37°C for 20 min, fixed with ice-cold methanol for 10 min, and then washed with ice-cold PBS. After blocking with 3% BSA, 0.25% Triton X-100, and PBS at RT for 1 h, samples were incubated with DyLight 488 anti-perforin mAb (1:200) or rabbit anti-granzyme B pAb (1:200) followed by Alexa Flour 546-conjugated donkey anti-rabbit antibody (1:200, Invitrogen) in the presence of 1% BSA at RT for 1 h. Cells were visualized with a Zeiss LSM510 Axiovert 200 M laser-scanning confocal microscope. The images were obtained using 63× Zeiss Plan-Aprochromat objective and LSM510 (version 7.1). To check for co-localization of CD38 and TRPM2, NK cells were incubated with B16F10 cells for indicated time periods, and fixed with ice-cold methanol for 10 min. After blocking with 3% BSA, 0.25% Triton X-100, and PBS at RT for 1 h, samples were incubated with goat anti-CD38 pAb (1:100, Santa Cruz Biotechnology) followed by Alexa Flour 488-conjugated donkey anti-goat antibody (1:200, Invitrogen) and DyLight 550 anti-TRPM2 (1:100, Abcam) pAb at RT for 1 h.

### Statistical analysis

Data are represented as the mean ± SEM of at least three independent experiments. Statistical analysis was performed using Student's *t*-test or one-way ANOVA as appropriate. *P* < 0.05 was considered significant.

## Author Contributions

S.-Y.R., J.-Y.K. and U.-H.K. designed research; S.-Y.R. and Y.-J.C. performed research; S.-Y.R. and U.-H.K. analyzed data; and S.-Y.R. and U.-H.K. wrote the paper. All authors discussed the results and commented on the manuscript.

## Supplementary Material

Supplementary Information Supplementary Information
