# Supplementary material for: ADP-ribose/TRPM2-mediated Ca2+ signaling is essential for cytolytic degranulation and antitumor activity of natural killer cells
Source: Sci Rep. 2015 Mar 25;5:9482. doi: 10.1038/srep09482 (PMC4399500; doi:10.1038/srep09482)
Supplement: Supplementary Information [file srep09482-s1.pdf]

Supplementary information

ADP-ribose and TRPM2-mediated Ca^{2+} signaling is essential for cytolytic degranulation and antitumor activity of natural killer cells

So-Young Rah,[1,2*] Jae-Yong Kwak,[2,4,5*] Yun-Jo Chung,[2] and Uh-Hyun Kim[1-3]

[1]Department of Biochemistry, [2]National Creative Research Laboratory for Ca^{2+} signaling Network, [3]Institute of Cardiovascular Research, [4]Division of Hematology and Oncology, Department of Internal Medicine, and [5]Research Institute of Clinical Medicine, Chonbuk National University Medical School, Jeonju, Republic of Korea

* These authors contributed equally to this study.

Supplementary Figure 1

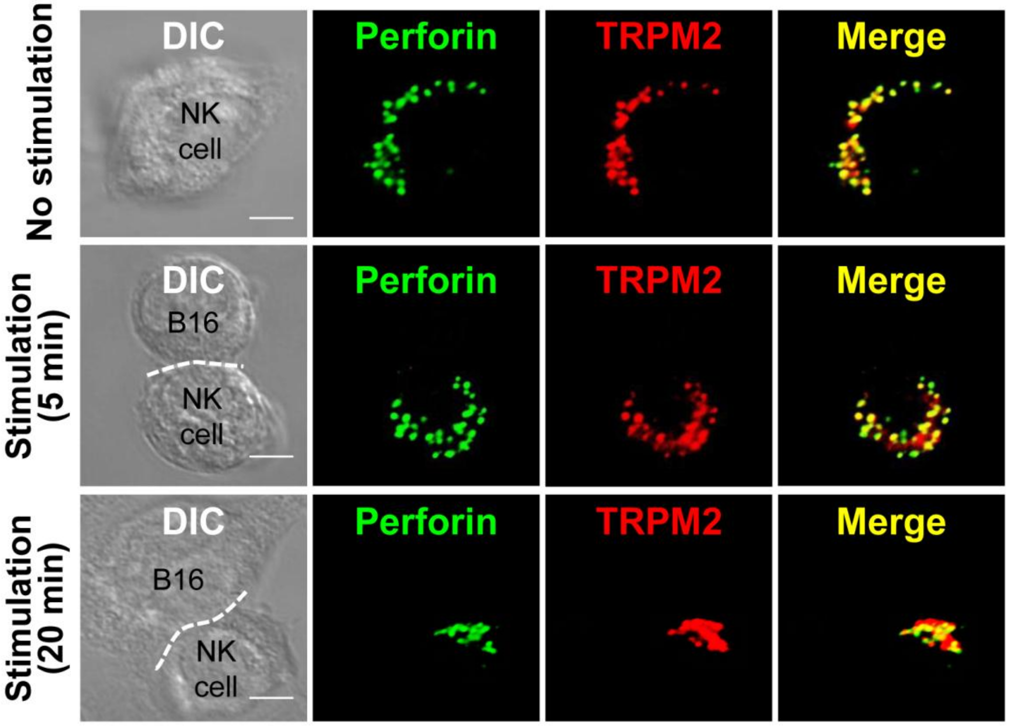

Figure S1. Tumor cell induces translocation of perforin and TRPM2 towards immunological synapses in NK cell. NK cells were added to B16F10 target cells at 1:1 ratio at 37℃ for indicated time and then stained with perforin and TRPM2. The dashed line indicates the immunological synapse. (Scale bar, 5 μm). All images were representative of at least three independent experiments.

Supplementary Figure 2

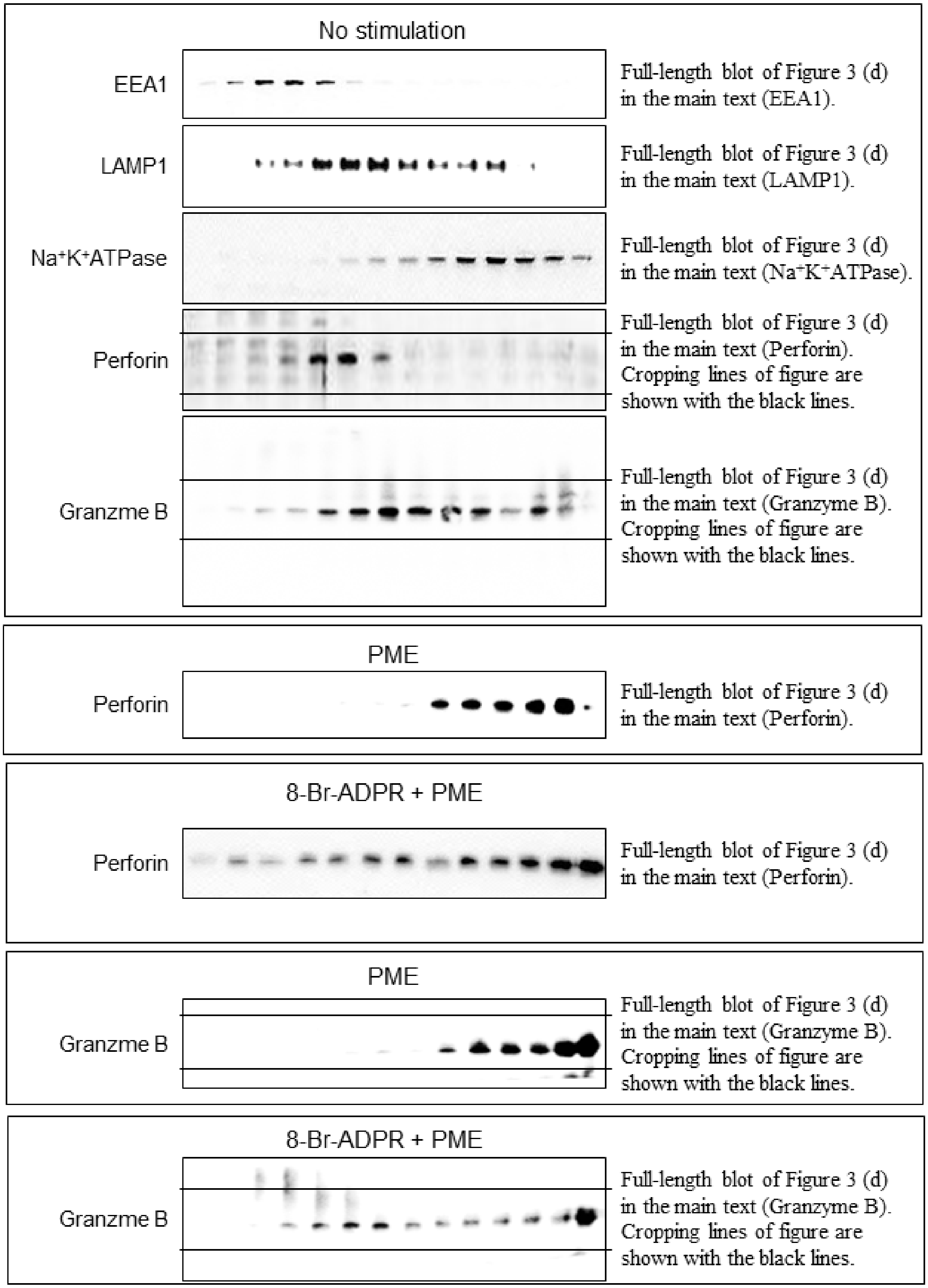

Supplementary Figure 3

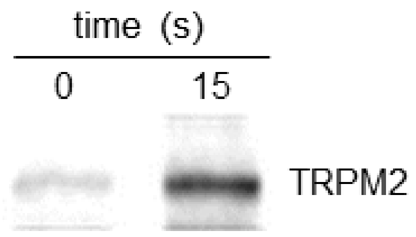

Full-length blot of Figure 6 (b, upper) in the main text (TRPM2).

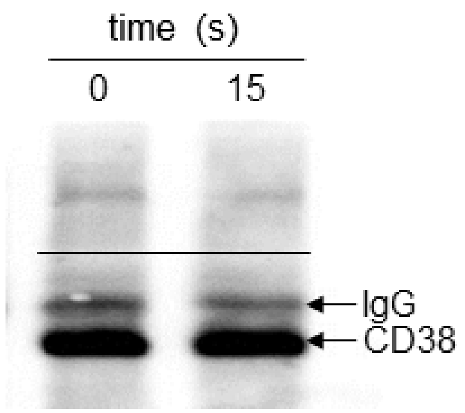

Full-length blot of Figure 6 (b, lower) in the main text (CD38).
Figure cropped below the black line.

Supplementary Figure 4

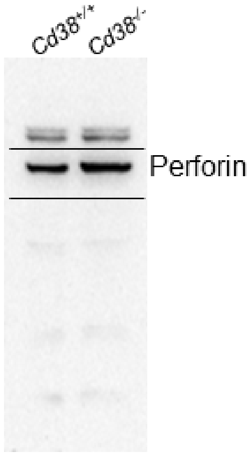

Full-length blot of Figure 7 (d) in the main text (Perforin). Cropping lines of figure are shown with the black lines.

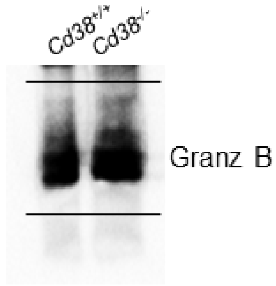

Full-length blot of Figure 7 (d) in the main text (Granzyme B). Cropping lines of figure are shown with the black lines.

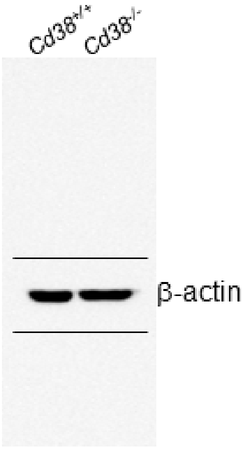

Full-length blot of Figure 7 (d) in the main text (β-actin). Cropping lines of figure are shown with the black lines.
